# Genome-Wide Analysis of the *GW2*-Like Genes in *Gossypium* and Functional Characterization of the Seed Size Effect of *GhGW2-2D*

**DOI:** 10.3389/fpls.2022.860922

**Published:** 2022-03-07

**Authors:** Li Huang, Shuxian Yang, Luyao Wu, Yue Xin, Jikun Song, Li Wang, Wenfeng Pei, Man Wu, Jiwen Yu, Xiaoyan Ma, Shoulin Hu

**Affiliations:** ^1^College of Plant Sciences, Tarim University, Xinjiang, China; ^2^State Key Laboratory of Cotton Biology, Key Laboratory of Cotton Genetic Improvement, Ministry of Agriculture, Institute of Cotton Research of Chinese Academy of Agricultural Sciences, Anyang, China; ^3^Western Agriculture Research Centre, Chinese Academy of Agricultural Sciences, Changji, China

**Keywords:** cotton, *GW2*-like, expression pattern, seed size, *GhGW2-2D*

## Abstract

Cotton is one of the most economically important crops worldwide. Seed size is a vital trait for plants connected with yield and germination. *GW2* encodes a RING_Ubox E3 ubiquitin ligase that controls seed development by affecting cell growth. Here, are few reports on *GW2*-like genes in cotton, and the function of *GW2* in cotton is poorly understood. In the present study, a genome-wide analysis identified 6 and 3 *GW2-*like genes in each of the two cultivated tetraploids (*Gossypium hirsutum* and *G. barbadense*) and each of their diploid ancestral species (*G. arboreum*, *G. raimondii*), respectively. *GhGW2-2D* has the same functional domain and high sequence similarity with *AtDA2* in *Arabidopsis*. Overexpression of *GhGW2-2D* in *Arabidopsis* significantly reduced seed and seedling size, suggesting *GhGW2-2D* is a potential target for regulating cotton seed size. These results provided information on the genetic and molecular basis of *GW2*-like genes in cotton, thus establishing a foundation for functional studies of cotton seeds.

## Introduction

Cotton is one of the most valuable commercial crops worldwide. Cotton fiber is important for textile manufacturing, and cottonseed is a major source of nutrients in human and livestock feed (Bellaloui et al., 2015; Bellaloui et al., 2021). Despite cottonseeds being of high economic value, they have not received sufficient attention in the past years (Li et al., 2009). Understanding the characteristics of cottonseeds is important for optimizing cotton plant growth, for instance, large seeds usually have higher germination and seedling vigor indices (Bewley, 1997; Pahlavani et al., 2008; Zhao et al., 2019). Seed size is an important trait that is limited by both genetic and environmental (Hu et al., 2021). Numerous studies conducted in rice (*Oryza sativa*) and *Arabidopsis thaliana* have provided an insight into the molecular mechanisms controlling seed size (Xing and Zhang, 2010; Valluru et al., 2014; Li et al., 2019). These mechanisms include several signaling pathways that control seed size, such as G-protein signaling, the ubiquitin-proteasome pathway, mitogen-activated protein kinase (MAPK) signaling, the HAIKU (IKU) pathway, phytohormones, and some transcriptional regulators (Li and Li, 2016; Li et al., 2019). The ubiquitin proteasome pathway plays an important role in controlling seed size (Li and Li, 2016; Li et al., 2019; Hao et al., 2021). Several RING-type E3 ubiquitin ligases have been reported to control seed size. For example, *Grain Width 2* (*GW2*), encoding RING_Ubox type E3 ubiquitin ligase, was found to affect grain size and improve grain yield potential (Song et al., 2007). GW2 directly interacts with EXPANSIN-like1 (EXPLA1), a cell wall-loosening protein that increases cell growth, and inactivates it through ubiquitination, thereby reducing seed growth (Choi et al., 2018). The function of *GW2*-like genes in plants has been reported in many types of crops. In spikelet hulls, a loss-of-function mutation in *GW2* has been reported to increase cell proliferation, leading to wide and heavy grains with a high yield of rice (Yamaguchi et al., 2020). Seeds of *GW2*-knockout mutants have a high protein content and essential dietary minerals in the endosperm (Achary and Reddy, 2021). Reducing the abundance of *GW2* transcript with RNA interference in the durum wheat cultivar Svevo increased the grain starch content, width, and surface area (Sestili et al., 2019). In addition, *GW2* homologs were also reported to control grain size in maize and wheat (Li et al., 2010; Su et al., 2011). The gene *AtDA2* shares significant homology with *OsGW2* (Xia et al., 2013), which encodes a protein that can monoubiquitinate *DA1* to activate its peptidase activity, thereby causing the cleavage of *DA1* substrates (Dong et al., 2017). In *Arabidopsis*, *AtDA2* also interacts with *AtDA1* and *da2-1* has larger seeds and organs than wild type, suggesting a conserved function between *OsGW2* and *AtDA2* in seed size control (Xia et al., 2013).

Nonetheless, the function of *GW2* in cotton is still poorly understood. In recent years, complete sequencing of the cotton genome has facilitated the comprehensive identification and analysis of the cotton genes (Wang K. et al., 2012; Li et al., 2014, 2015; Zhu et al., 2017). In this study, we identified several *GW2*-like genes in *Gossypium hirsutum* (6), *G. barbadense* (6), *G. arboreum* (3), and *G. raimondii* (3). The sequence characteristics, chromosomal distribution, evolutionary relationship, and the expression patterns of *GW2*-like genes in cotton were analyzed. As the homologous gene of *AtDA2* and *OsGW2*, *GhGW2-2D* was also found to encode a RING_Ubox domain in cotton, which may negatively regulate seed size. Then we overexpressed *GhGW2-2D* into *Arabidopsis thaliana* ecotype Col-0 to further investigate the function of *GhGW2-2D*. The results of this study lay the foundation for future studies on *GW2-*like genes in the improvement of cotton seed size.

## Materials and Methods

### Sequence Retrieval and Identification of *GW2*-Like Genes in Cotton

The four completed genome assemblies of *G. arboreum* (A2, CRI_V1.0) (Du et al., 2018), *G. raimondii* (D5, JGI v2_a2.1) (Paterson et al., 2012), *hirsutum acc.* TM-1 (AD1, ZJU) (Hu et al., 2019), and *G. barbadense acc.*H7124 (AD2, ZJU) (Hu et al., 2019) were downloaded from the CottonGen database^1^ (Yu et al., 2014). The protein sequences of AtDA2 and OsGW2 were acquired from the *Arabidopsis* Information Resource^2^ and the Rice Genome Annotation Project^3^, respectively. The protein sequences of AtDA2 and OsGW2 were used as queries to identify *GW2*-like genes against four genome databases of *Gossypium* with *e*-values of 1e^––10^. The candidate sequences were submitted to the NCBI batch CDD program^4^ and SMART database^5^ (Letunic et al., 2015) for further confirmation.

### Chromosomal Location

The chromosomal distribution and the loci of all *GW2*-like genes were determined based on the results of identification genome annotation data. Molecular markers of reported QTLs were download from CottonQTLdb^6^ (Said et al., 2013, 2015a,b). Subsequently, the location images of *GW2*-like genes were drawn using the MapChart software (Voorrips, 2002).

### Phylogenetic Analysis

The phylogenetic tree of *GW2*-like genes was analyzed and generated using multiple sequence alignments of four cultivated cotton species using ClusterW^7^ (Larkin et al., 2007). A phylogenetic tree was constructed using MEGA 7.0^8^ (Kumar et al., 2016) with pairwise distance and the neighbor-joining (NJ) method.

### Gene Duplication and Synteny Analysis

Gene duplication events and genomic synteny were analyzed using MCScanX and visualized using the TBtools software (Wang Y. et al., 2012; Cheng et al., 2020). The synonymous (Ks) and non-synonymous (Ka) substitution ratios were calculated using TBtools software.

### Gene Structure Analysis and Identified Motifs

The structural information of GFF3 files of four cotton species were download from CottonGen database (see text footnote 1). The protein length, molecular weight, and isoelectric point of the encoded proteins were predicted using the ProtParam tool^9^ (Gasteiger et al., 2003). To clarify the conserved motifs and protein domains, the online program MEME^10^ (Bailey et al., 2009) and NCBI database were used, respectively. The structure of *GW2*-like genes was predicted using TBtools software (Cheng et al., 2020).

### Plant Material, RNA Extraction, and qRT-PCR Analysis

Wild-type cotton (TM-1) and *Arabidopsis* (Col-0) were planted in the experimental field and greenhouse of the Institute of Cotton Research of Chinese Academy of Agricultural Sciences under conventional field management conditions in Anyang, China. To detect the relative expression of *GW2*-like genes, different samples were collected from WT cotton at different stages of ovule development. All ovule samples were self-pollinated. Total RNA was isolated from samples using Fast Pure Plant Total RNA Isolation Kit (Vazyme, Nanjing, China). The qRT-PCR analysis primers were listed in Supplementary Table 1. Gh_D03G0370 (*GhActin3*) (Li et al., 2005) and AT3G18780 (*AtActin2*) (Wang et al., 2016) were used as internal controls for the qPCR experiments.

### Constructs and Transformation

The 1,272 bp complete coding sequence (CDS) of *GhGW2-2D* was amplified using the primers *35S:GhGW2-2D*-F and *35S:GhGW2-2D*-R (Supplementary Table 1). The *35S:GhGW2-2D* construct were cloned into a pBI121 vector digested with *Bam*HI and *Sac*I using a PCR-based homologous recombination system. The *35S:GhGW2-2D* plasmid transferred into Col-0 plants using *Agrobacterium* GV3101 and the transformants were selected on medium supplemented with kanamycin (50 mg/L). Three homozygous T_3_ generation lines were used for further analysis. The seed and cotyledon were conducted by scanning to generate a digital image and then measured by ImageJ software.

## Results

### Identification of *GW2*-Like Genes in *Gossypium*

In order to identify all GW2-like proteins in tetraploid cotton (*G. hirsutum* and *G. barbadense*) and their diploid ancestors (*G. arboreum* and *G. raimondii*), the protein sequences of AtDA2 and OsGW2 in rice and *Arabidopsis* were used as queries. We identified three, three, six, and six *GW2*-like genes in *G. arboreum*, *G. raimondii*, *G. barbadense*, and *G. hirsutum*, respectively. The length of these cotton *GW2*-like gene sequences ranged from 1,260 to 1,290 bp, encoding polypeptides from 419 to 429 amino acids long. The predicted isoelectric point of proteins varied from 4.70 to 4.93. According to the results of evolutionary analysis, all members were divided into three subgroups. More details of the gene names, locus IDs, and other features are shown in Table 1.

**TABLE 1:** Information of the *GW2-*like genes in *Gossypium*.

Group name	Gene name	Gene identifier	Chromosomal localization	pI	MW (KD)	Size (AA)
Group 1	Ga01G0386	*GaGW2-1*	A01	4.90	47.58	429
	GB_A01G0356	*GbGW2-1A*	A01	4.93	47.45	428
	GB_D01G0377	*GbGW2-1D*	D01	4.83	47.5	429
	GH_A01G0369	*GhGW2-1A*	A01	4.93	47.52	429
	GH_D01G0356	*GhGW2-1D*	D01	4.82	47.52	429
	Gorai.002G038700	*GrGW2-1*	D01	4.83	47.53	429
Group 2	Ga05G3697	*GaGW2-2*	A05	4.84	46.97	423
	GB_A04G0530	*GbGW2-2A*	A04	4.85	47.08	423
	GB_D05G3554	*GbGW2-2D*	D05	4.82	46.97	423
	GH_A04G0522	*GhGW2-2A*	A04	4.85	46.91	423
	GH_D05G3532	*GhGW2-2D*	D05	4.79	46.61	419
	Gorai.009G359400	*GrGW2-2*	D05	4.82	46.96	423
Group 3	Ga13G1904	*GaGW2-3*	A13	4.85	47.02	426
	GB_A13G1898	*GbGW2-3A*	A13	4.88	46.75	423
	GB_D13G1839	*GbGW2-3D*	D13	4.73	47.25	428
	GH_A13G1787	*GhGW2-3A*	A13	4.85	46.73	423
	GH_D13G1740	*GhGW2-3D*	D13	4.73	47.31	429
	Gorai.013G177000	*GrGW2-3*	D13	4.70	47.26	429

### Chromosomal Location of *GW2-*Like Genes

All *GW2-*like genes were unevenly mapped onto chromosomes based on the genome annotation data of the four species (Supplementary Figure 1 and Table 1). In the *G. arboreum* genome, the three identified *GaGW2s* were three located on chromosomes A01, A05, and A13. In *G. raimondii*, the three *GrGW2* genes were distributed on three chromosomes: chromosome 02 (D01), chromosome 09 (D05), and chromosome 13 (D13). In *G. hirsutum*, six *GhGW2* genes were mapped on three At subgenome chromosomes (A01, A05, and A13) and three Dt subgenome chromosomes (D01, D05, and D13). The chromosomal distribution of *GW2*-like genes in the *G. barbadense* genome was resemble to that of *GhGW2s*. Therefore, it could infer that *GW2*-like genes were highly conserved in the evolution from diploid to tetraploid in cotton.

### Gene Structure and Protein Domain Analysis of *GW2*-Like Genes

An efficient way to predict gene function is to analyze its gene structure and the encoded protein domains. In this study, the *GW2*-like gene structures of four cotton species were visualized to further understand their differences and potential functional diversity.

**FIGURE 1:**
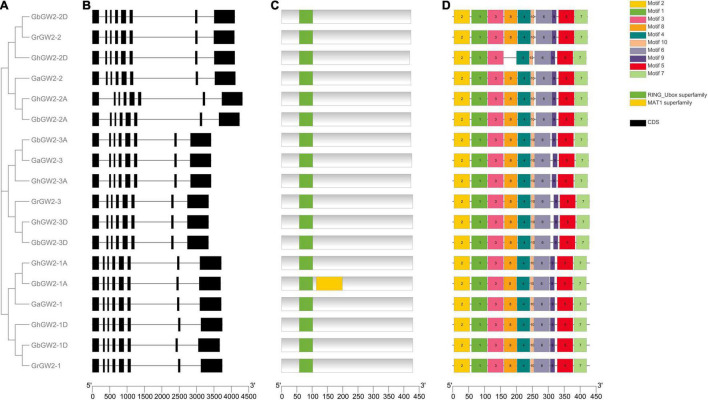
Phylogenetic analysis, gene structure, and protein domain analyses of *GW2-*like genes in *Gossypium*. **(A)** Phylogenetic tree of *GW2-*like genes in *Gossypium*. Phylogenetic tree was constructed using MEGA 7.0 by Neighbor-Joining method with 1,000 bootstrap replicates. **(B)** The exon-intron structure of *GW2-*like genes. **(C)** GW2-like protein domain prediction. **(D)** The motifs prediction of GW2-like protein.

A phylogenetic tree of GW2-like genes in *G. hirsutum* and *G. barbadense* and their ancestral diploids was constructed using the NJ method (Figure 1A). All GW2-like proteins were divided into three subgroups. Each group contained six members, one from *G. arboreum*, one from *G. raimondii*, two from *G. hirsutum*, and two from *G. barbadense*. All *GW2*-like genes have eight exons that were highly conserved in the four species of cotton (Figure 1B).

The NCBI and SMART databases were used to predict the domains of the GW2-like proteins. All GW2-like proteins had a typical E3 ubiquitin ligase RING_Ubox domain (Figure 1C), which can bind to a specific E2 ubiquitin-binding enzyme and determines the specificity of the ubiquitinated substrate. The MEME online program was used to predict the motifs of the GW2-like family genes. The results showed that most members in the same subgroup had resemble gene structures and functional domains (Figure 1D).

### Adaptive Evolution Analysis of the *GW2*-Like Genes

**FIGURE 2:**
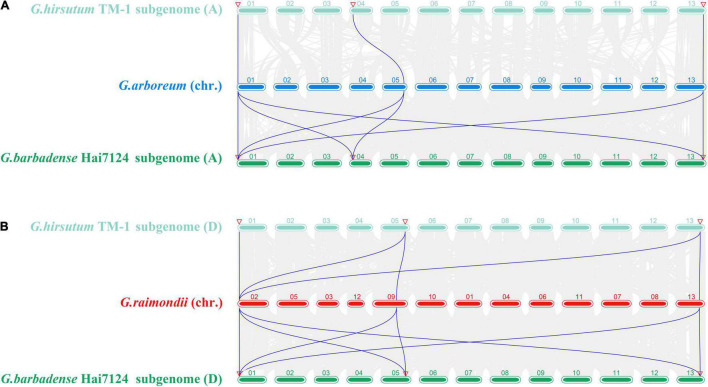
Analysis of synteny among multiple *Gossypium* genomes about *GW2*-like. **(A)** Synteny analysis among *G. arboreum*, *G. hirsutum* (At subgenome), and *G. barbadense* (At subgenome). **(B)** Synteny analysis among *G. raimondii*, *G. hirsutum* (Dt subgenome), and *G. barbadense* (Dt subgenome).

Gene replication and subsequent functionalization are important driving forces for genome and species evolution. In addition, gene duplication events are important for amplification of gene families. To clarify the amplification mechanism and evolution of *GW2*-like genes in the four cotton species, we conducted interspecific collinearity analysis and gene repeat event analysis (Figure 2 and Table 2).

**TABLE 2:** Ka and Ks calculations for *GW2-*like duplicated gene pairs.

Species	Gene 1	Gene 2	Ka	Ks	Ka/Ks	Duplicate type
*G. hirsutum*	*GhGW2-1A*	*GhGW2-2A*	0.14	0.48	0.29	Segmental
	*GhGW2-1A*	*GhGW2-3A*	0.15	0.47	0.32	Segmental
	*GhGW2-1A*	*GhGW2-1D*	0.01	0.05	0.27	Segmental
	*GhGW2-1A*	*GhGW2-2D*	0.14	0.46	0.29	Segmental
	*GhGW2-1A*	*GhGW2-3D*	0.15	0.41	0.37	Segmental
	*GhGW2-2A*	*GhGW2-1D*	0.15	0.44	0.33	Segmental
	*GhGW2-2A*	*GhGW2-2D*	0.01	0.06	0.18	Segmental
	*GhGW2-3A*	*GhGW2-3D*	0.01	0.07	0.16	Segmental
	*GhGW2-1D*	*GhGW2-2D*	0.14	0.42	0.33	Segmental
	*GhGW2-1D*	*GhGW2-3D*	0.15	0.37	0.41	Segmental
*G. barbadense*	*GbGW2-1A*	*GbGW2-2D*	0.14	0.47	0.29	Segmental
	*GbGW2-1A*	*GbGW2-2A*	0.14	0.49	0.29	Segmental
	*GbGW2-1A*	*GbGW2-3D*	0.15	0.41	0.38	Segmental
	*GbGW2-1A*	*GbGW2-1D*	0.02	0.05	0.33	Segmental
	*GbGW2-2A*	*GbGW2-2D*	0.01	0.06	0.22	Segmental
	*GbGW2-2A*	*GbGW2-1D*	0.15	0.44	0.33	Segmental
	*GbGW2-3A*	*GbGW2-3D*	0.01	0.06	0.21	Segmental
	*GbGW2-3A*	*GbGW2-1D*	0.15	0.42	0.37	Segmental
	*GbGW2-1D*	*GbGW2-2D*	0.14	0.42	0.34	Segmental
	*GbGW2-1D*	*GbGW2-3D*	0.15	0.36	0.42	Segmental

Collinearity analysis was performed for *G. arboreum* and *G. hirsutum* (A subgenome), *G. arboreum* and *G. barbadense* (A subgenome), *G. raimondii* and *G. hirsutum* (D subgenome), *G. raimondii* and *G. barbadense* (D subgenome), respectively (Figure 2). A total of three, six, five, and seven pairs of collinear genes were found, respectively. The *GbGW2*-like genes in *G. barbadense* had more collinear gene pairs with *G. arboreum* (A2) and *G. raimondii* (D5), while the *GhGW2*-like genes in *G. hirsutum* had fewer collinear gene pairs with *G. arboreum* (A2) and *G. raimondii* (D5), indicating that *GW2*-like genes in *G. barbadense* were more related to two possible ancestral species of *G. arboreum* and *G. raimondii* than *GW2*-like genes in *G. hirsutum*.

In order to elucidating the evolutionary dynamics and selection pressure of protein coding, the non-synonymous (Ka) and synonymous (Ks) replacement rates were calculated using full-length sequences between each pair of linear homologous genes. Generally, a Ka/Ks ratio>1, = 1 and <1 indicates diversified selection, neutral selection, and purification selection, respectively. The Ka/Ks ratio of all gene pairs was less than 1, suggesting that these genes had undergone purification selection (Table 2). Above results indicated that the *GW2*-like genes in *G. hirsutum* and *G. barbadense* were relatively conserved.

### Expression Profiles of *GhGW2*-Like Genes in Cotton

**FIGURE 3:**
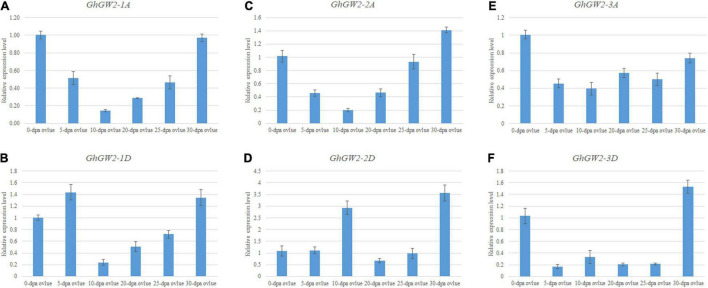
qRT-PCR of Gh*GW2*s in ovules. Relative expression level of *GhGW2-1A* **(A)**, *GhGW2-1D* **(B)**, *GhGW2-2A* **(C)**, *GhGW2-2D* **(D)**, *GhGW2-3A* **(E)**, *GhGW2-3D* **(F)**. GhActin3 was selected as internal and the 1 Ct value of 0-DPA-ovules was set as the control. The data presented are the means ± SD of three replicates.

To investigate the tissue-specific expression profiles of *GW2*-like genes, published TM-1 expression data, including root, stem, leaf, torus, petal, stamen, pistil, calycle, fiber and ovule at various developmental stages were used. Most *GhGW2* genes were extensively expressed in many tissues (Supplementary Figure 2). *GhGW2-1A* and *GhGW2-1D* were relatively highly expressed in pollen, petal, and pistil. *GhGW2-2A* was highly expressed in 20 DPA fiber and roots. *GhGW2-2D* was highly expressed in the stem, root and early stages of ovule. *GhGW2-3A* and *GhGW2-3D* had similar expression trends with *GhGW2-1A* that highly expressing in flowers and pistils (Supplementary Figure 2A). The expression patterns of *GbGW2*-like genes in *G. barbadense* and *G. hirsutum* shared alike (Supplementary Figure 2B), indicating that these genes may have similar functions. qRT-PCR of diffident ovule development stages showed that *GhGW2-1A*, *GhGW2-2A* and *GhGW2-1D* had a similar tendency with highly expressed in early stages and later stages, and less expressed in 10–20 DPA ovules (Figures 3A–C). *GhGW2-2D* expression was highest in 10 DPA ovule and 30 DPA ovule, suggesting it may involve in cotton ovules development (Figure 3D). *GhGW2-3A* revealed an extensive expression in all ovule development stages (Figure 3E). *GhGW2-3D* was expressed at very low levels in all ovule development stages except in 30 DPA ovule (Figure 3F).

*GhGW2*-like genes had distinct expression patterns in different tissues of TM-1, suggesting that *GhGW2*-like genes may have different functions in cotton growth.

### Generation of *GhGW2-2D*-Overexpressing *Arabidopsis* Lines

**FIGURE 4:**
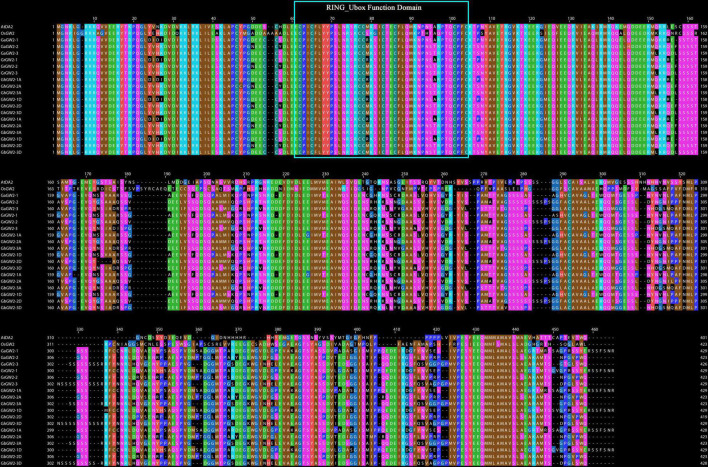
Multiple sequence alignments of GW2-like amino acid sequences.

**FIGURE 5:**
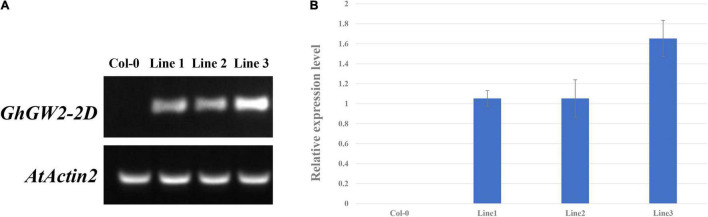
**(A)** Identification of *GhGW2-2D* transgenic plants using semi-quantitative PCR. **(B)** Relative expression level of *GhGW2-2D* in three transgenic *Arabidopsis* lines. The Ct value of *GhGW2-2D* in transgenic line 1 was set as the control. The data presented are the means ± SD of three replicates.

Based on the sequence alignment and homology analysis, *GhGW2-2D* was selected for further study because of its similarity to *OsGW2* and *AtDA2* (Figure 4). Moreover, the expression of *GhGW2-2D* was relatively high in the stage of ovule development. Plants overexpressing *GhGW2-2D* were generated and identified using semi-quantitative PCR (Figure 5A), and a total of three transgenic lines with high expression of *GhGW2-2D* were identified. qRT-PCR was used to further assess the relative expression levels of the three diverse transgenic lines (Figure 5B).

### Overexpression of *GhGW2-2D* Decreases Seed Size

**FIGURE 6:**
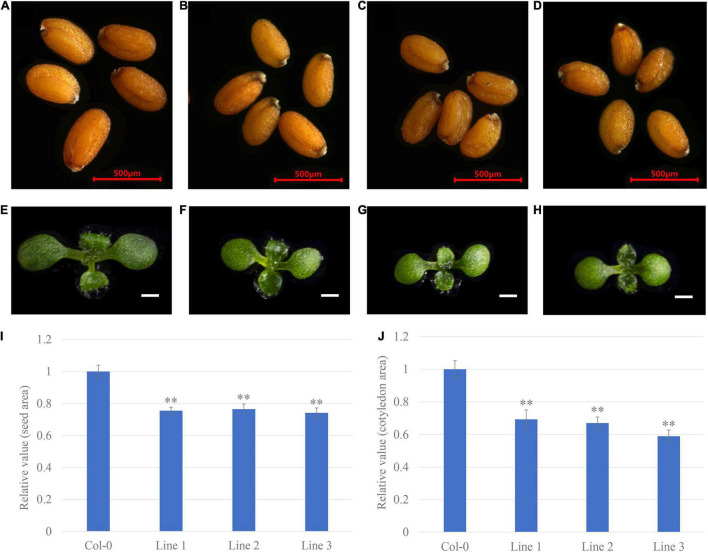
The overexpression of *GhGW2-2D* decreases the seed size in *Arabidopsis*. **(A–D)** Seed phenotype of Col-0 **(A)** and transgenic lines 1 **(B)**, 2 **(C)**, and 3 **(D)** (*n* = 30). Bar = 500 μm. **(E–H)** Seedling phenotype of Col-0 **(E)** and transgenic lines 1 **(F)**, 2 **(G)**, and 3 **(H)** (*n* = 20). Bar = 1 mm. The relative seed **(I)** and cotyledon **(J)** area of seeds from transgenic lines compared to Col-0. Values in **(I)** and **(J)** are given as mean ± SE relative to the respective Col-0 values, set at 1. ** represents significant differences at the *P* < 0.01 level.

To evaluate the applicability of transgenic breeding to alter seed size, different developmental stages of transgenic *Arabidopsis* were characterised. Overexpression of *GhGW2-2D* reduced seed size (Figures 6A–D). The seed areas of lines 1, 2, and 3 decreased by 25, 24, and 26%, respectively, compared with that of the wild type Col-0 (Figure 6I). The growth of nine-day-old seedlings was measured after germinating. The transgenic lines had smaller cotyledons than Col-0 (Figures 6E–H). Compared with that of wild type Col-0, the cotyledon area of lines 1, 2, and 3 decreased by 31, 33, and 42%, respectively (Figure 6J). These results further support the role of *GhGW2-2D* in limiting seed and organ growth.

## Discussion

Seed size is a vital characteristic of flowering plants (Orsi and Tanksley, 2009). Cottonseed kernels are regarded as the best source of vegetable protein and oil and are associated with seed size (Poehlman, 1994; Pahlavani et al., 2009). Advances have revealed that plants with large seeds accumulate more nutrients, which may affect seed germination, plant growth, and development (Wang, 2008). Thus, understanding the mechanisms controlling seed size is essential for agricultural production and germplasm improvement.

Recent studies have shown that *GW2*-like genes have a far-reaching impact on seed size and weight. *GW2*, which encodes an E3 ubiquitin ligase involved in the regulation of cell division, was found to affect grain size and improve grain yield potential (Song et al., 2007). The seeds of the offspring of mutant *da2-1* plants were bigger than those of the control, and the 1,000 grain weight was increased (Xia et al., 2013). Moreover, the loss of the function of this gene has been reported to increase the number of cells and the grain size of rice (Yamaguchi et al., 2020). Altogether, these studies highlight that the *GW2* gene has an important value in breeding and plays an important regulatory role in controlling seed development.

We identified 3, 3, 6, and 6 *GW2-*like genes in *G. raimondii*, *G. arboreum, G. barbadense*, and *G. hirsutum*, respectively. The chromosomal distribution, evolutionary relationship, and expression patterns of *GW2*-like genes in cotton were analyzed. It has been found that all *GW2-like* genes have a typical RING_U-box domain, which can interact with a specific E2 ubiquitin binding enzyme and ubiquitinate specific substrates (Song et al., 2007). Gene structure analysis revealed that all *GW2-*like genes have eight exons, which indicates that the gene may be functionally conserved during evolution. According to the selection pressure analysis, the Ka/Ks ratio of *GW2*-like was less than 1, further supporting the evolutionary conservation of these genes. The GhGW2-2D was co-localized with quantitative trait loci (QTL) for seed size (boll weight, BW), suggesting that its sequence variations may be genetically associated with the natural variation in seed size (Supplementary Figure 3). Processing and analysis of published RNA-Seq data, we found that *GhGW2-2D* was highly expressed in roots, stems, and during early ovule development. Moreover, GhGW2-2D is most similar to AtDA2 and OsGW2 and has the same protein conserved functional domain. Therefore, this gene was selected for the subsequent analysis.

To date, only few studies have investigated *GW2-*like genes in cotton. In the present study, a comprehensive analysis of *GW2-*like genes in the four sequenced cotton species was performed, which provided more details of the cotton *GW2-*like genes. Overexpression of *GhGW2-2D* in *Arabidopsis* decreased seed size, indicating that the product encoded by this gene may play a negative regulatory role in seed development. *DA1* interacts with the E3 ubiquitin ligase *DA2* to regulate seed and organ size in *Arabidopsis*, *Triticum aestivum*, and *G. hirsutum* (Xia et al., 2013; Liu et al., 2020; Yang et al., 2021). It may be interesting to further explore the molecular network controlling seed size in cotton. These findings could provide an important theoretical and experimental basis for growth and regulating the development of seeds and organs in cotton, which has great significance in creating excellent germplasm.

## Data Availability Statement

The original contributions presented in the study are included in the article/Supplementary Material, further inquiries can be directed to the corresponding authors.

## Author Contributions

LH and SY planned the experiments and wrote the manuscript. LWu and YX participated in the study. JS, MW, WP, and JY provided advice for experiments and manuscript writing. XM and SH conceived and designed the research and manuscript revision. All authors read and approved the final manuscript.

## Conflict of Interest

The authors declare that the research was conducted in the absence of any commercial or financial relationships that could be construed as a potential conflict of interest.

## Publisher’s Note

All claims expressed in this article are solely those of the authors and do not necessarily represent those of their affiliated organizations, or those of the publisher, the editors and the reviewers. Any product that may be evaluated in this article, or claim that may be made by its manufacturer, is not guaranteed or endorsed by the publisher.

## Footnotes

1
https://www.cottongen.org

2
http://www.arabidopsis.org

3
http://rice.uga.edu/expression.shtml

4
https://www.ncbi.nlm.nih.gov/Structure/bwrpsb/bwrpsb.cgi

5
http://smart.embl-heidelberg.de/

6
http://www2.cottonqtldb.org:8081/

7
http://www.ebi.ac.uk/Tools/msa/clustalw2

8
http://www.megasoftware.net

9
https://web.expasy.org/

10
http://meme-suite.org/

## Supplementary Material

Supplementary Figure 1 Chromosomal localization of *GW2-*like genes in *Gossypium*. All *GW2*-like genes were mapped onto chromosomes of *G. arboreum* **(A)**, *G. raimondii* **(B)**, *G. hirsutum* **(C)**, and *G. barbadense* **(D)**. The scale represents megabases (Mb).

Supplementary Figure 2 Expression profiles of *GW2*-like genes in *G. hirsutum* TM1 and *G. barbadense* Hai7124. **(A)** Expression patterns of *GhGW2*-like genes. **(B)** Expression patterns of *GbGW2*-like genes.

Supplementary Figure 3 A co-localization analysis of *GhGW2*-like genes with seed size quantitative trait loci (QTLs). *GhGW2*-like genes and co-localized QTL are shown in red.
