# Supplementary material for: Genome-Wide Analysis of the GW2-Like Genes in Gossypium and Functional Characterization of the Seed Size Effect of GhGW2-2D
Source: Front Plant Sci. 2022 Mar 7;13:860922. doi: 10.3389/fpls.2022.860922 (PMC8940273; doi:10.3389/fpls.2022.860922)
Supplement: Supplementary file 1 [file Table_1.DOCX]

**Additional file 1: Table S1.** Primers used in this paper.

| **Number** | **Name** | **Sequence (5′-3′)** |
| --- | --- | --- |
| 1 | CDS-GhGW2-F | ATGGGTAATAAGTTGGGAAG |
| 2 | CDS-GhGW2-R | CTATTGCCATGGAACTCCAGG |
| 3 | 35S::GhGW2-2D-F | GGACTCTAGAGGATCC ATGGGTAATAAGTTGGGAAGGAGA |
| 4 | 35S::GhGW2-2D-R | GGACTCTAGAGGATCC CTATTGCCATGGAACTCCAGG |
| 5 | qRT-GhGW2-1A-F | CACAACCTGCCTTGATGAGTAA |
| 6 | qRT-GhGW2-1A-R | TTCCGTGACCATTATGTCCTC |
| 7 | qRT-GhGW2-2A-F | GGAAGTTCAATACGGTTCAGC |
| 8 | qRT-GhGW2-2A-R | TCATCATCCCTGTTTGTCCT |
| 9 | qRT-GhGW2-3A-F | GGCGATGAAGAATGCTGTTC |
| 10 | qRT-GhGW2-3A-R | ACTGGGTAGGACGGGTTGAA |
| 11 | qRT-GhGW2-1D-F | CACAACCTGCCTTGATGAGTAA |
| 12 | qRT-GhGW2-1D-R | TTCCGTGACCATTATGTCCTC |
| 13 | qRT-GhGW2-2D-F | AACCTCAAACTACGCTGTGG |
| 14 | qRT-GhGW2-2D-R | GTTCCTGCTGCCTCATTCTA |
| 15 | qRT-GhGW2-2D-F | ATGGAAGCAGTTTGGCAGTC |
| 16 | qRT-GhGW2-2D-R | CAGAAGGAGATGATGATGAACC |
| 17 | At-Actin-F | AGAAACCCTCGTAGATTGGCAC |
| 18 | At-Actin-R | ACTCTCCCGCTATGTATGTCGC |
| 19 | Actin-F | ATCCTCCGTCTTGACCTTG |
| 20 | Actin-R | TGTCCGTCAGGCAACTCAT |
