# Supplementary material for: Genome-Wide Analysis of the GW2-Like Genes in Gossypium and Functional Characterization of the Seed Size Effect of GhGW2-2D
Source: Front Plant Sci. 2022 Mar 7;13:860922. doi: 10.3389/fpls.2022.860922 (PMC8940273; doi:10.3389/fpls.2022.860922)
Supplement: Supplementary file 2 [file Table_2.DOCX]

**Additional file 2:**

Coding sequence of *GhGW2-2D*

1 ATGGGTAATAAGTTGGGAAGGAGAAGGCAAGTGGTGGACGAGAAGTATACGCGCCCCCAA

61 GGGTTGTATGTTCATAAAGATGTGGATGTTAAGAAGCTGAGAAAACTGATACTTGAATCG

121 AAGCTTGCTCCATGTTACCCTGGCAATGAAGAGTGCTGTTATGATCTTGAAGAATGCCCA

181 ATTTGCTTTTTGTATTACCCGAGTCTCAACAGATCAAGATGTTGCATGAAAAGTATTTGC

241 ACAGAGTGTTTTCTACAGATGAAGAATCCAAACTCGACCCGTCCTACCCAGTGTCCTTTC

301 TGCAAAACCTCAAACTACGCTGTGGAGTACCGAGGTGTGAAAACAAAGGAGGAAAAAGGG

361 ATCGAGCAAATTGAAGAACAACGTGTCATAGAAGCACAAATTAGAATGAGGCAGCAGGAA

421 CTTCAGGATGACGAAGAGAGAATGCAGAAAAGACAAGAATTCAGTTCTTCAAGCACCGCT

481 GTTTCACCGGGGGAAGTTCAATACGGTACAGCTGCTGCTCAATCCTCTGTTGAGGAGGAA

541 CTAGTTTCTTCTCAAGATTCGCAGGCTGCCATGATGGTTCAACAACCATCACATCCTAGG

601 ACAAACAGGGATGATGAGTTTGACGTAGATCTGGAGGAAATAATGGTCATGGAAGCAATT

661 TGGCAGTCGATTCAGGAGAACAGCAGACACAGAAAGTCTAACAATGGAGATGCTGCTTCT

721 TCAGTACATGTTTCAGTAGATCGCTATGTCTCACCAGCTATGGCCACAGTGGCCGGTTCA

781 TCATCATCATCATCATCATCATCTCCTTCTGGTGGTTTTGCTTGTGCAGTAGCTGCCCTT

841 GCTGAGCGTCAGCAGATCAGTGGAGAATCTTCTCTTGACTACAATGGAAATATACCACCG

901 TTCAATATGCTTCCTGGCAGCAGCAGGTTTTATAACAGGTTGGACCCAGTTTCCGAGAAT

961 CGTCCTGCAGAGAGCCCGGTTGACATGCCAACTGGTGGTCTGATGACACCTTCAAGAGAT

1021 GAAGGGGAATGGGGAGTAGATTTTGGATCGGAGGTGGCTGAAGCAGGGACTAGCTACGCA

1081 AGTCCTGATGTTACAGAAGATATAGGCGGGATCTCAACAATACCACAACAGGATGAAATA

1141 AGGGGTAGCTTTCTAAATGTGCCCCGACCCATTGTTCCGGAAAGTTATGAAGAGCAGATG

1201 ATGCTGGCTATGGCTGTATCTCTGTCTGAAGCTAAAGCTATGACAAGTAACCCTGGAGTT

1261 CCATGGCAATAG
