# Supplementary figures and images for: Genome-Wide Analysis of the GW2-Like Genes in Gossypium and Functional Characterization of the Seed Size Effect of GhGW2-2D
Source: Front Plant Sci. 2022 Mar 7;13:860922. doi: 10.3389/fpls.2022.860922 (PMC8940273; doi:10.3389/fpls.2022.860922)

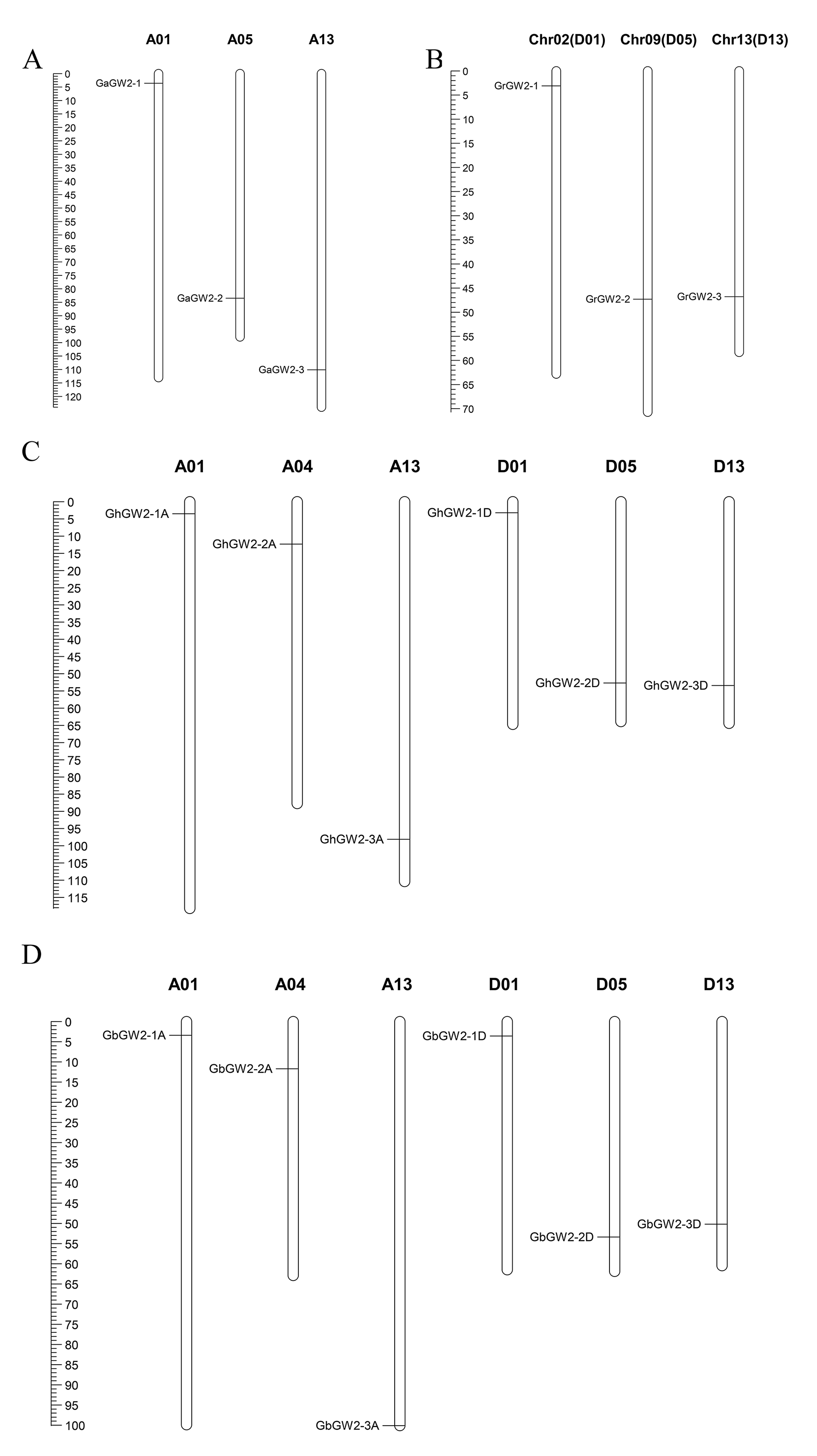

Supplement: Supplementary Figure 1 — Chromosomal localization of GW2-like genes in Gossypium. All GW2-like genes were mapped onto chromosomes of G. arboreum (A), G. raimondii (B), G. hirsutum (C), and G. barbadense (D). The scale represents megabases (Mb). [file Image_1.TIF]

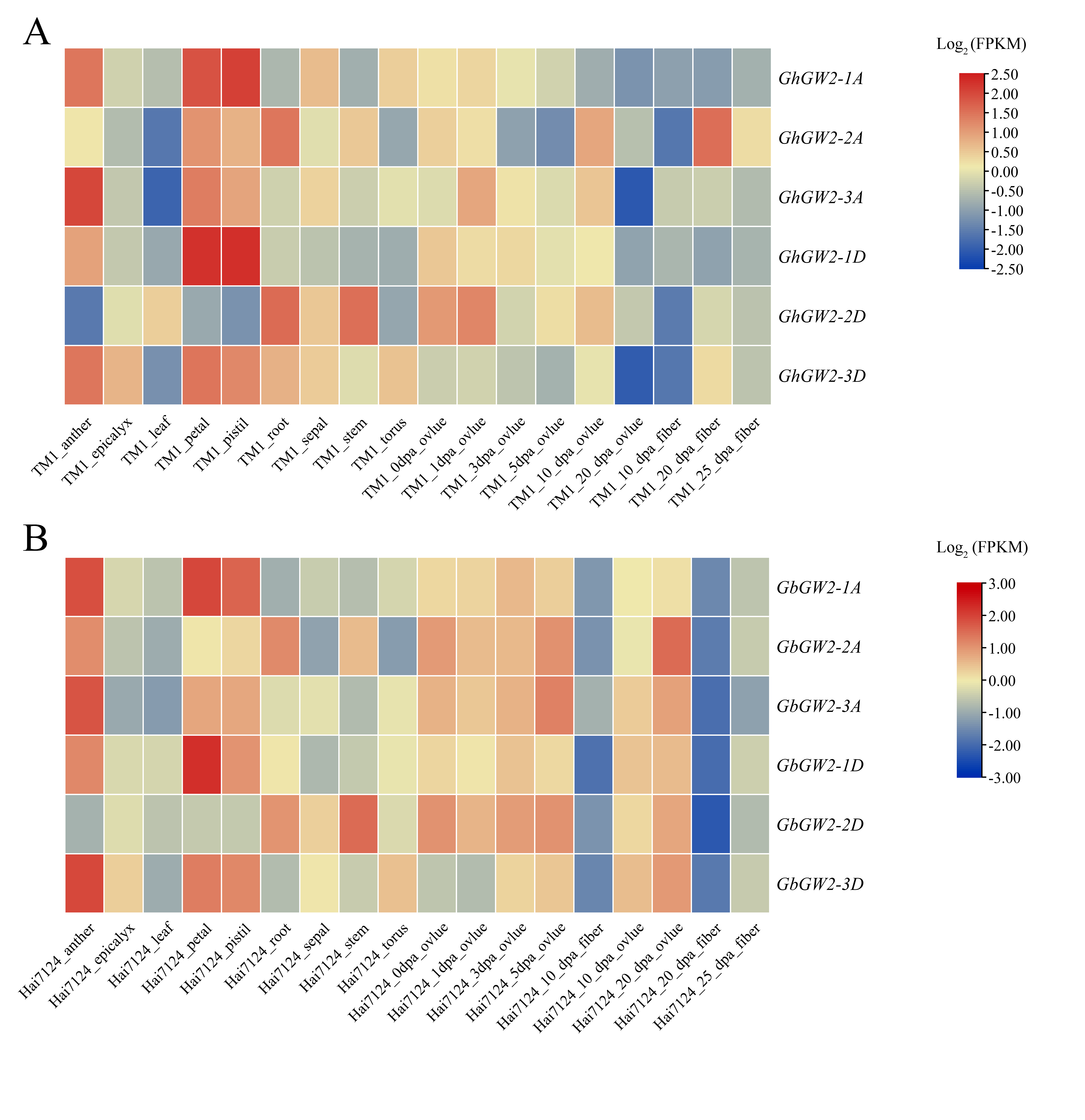

Supplement: Supplementary Figure 2 — Expression profiles of GW2-like genes in G. hirsutum TM1 and G. barbadense Hai7124. (A) Expression patterns of GhGW2-like genes. (B) Expression patterns of GbGW2-like genes. [file Image_2.TIF]

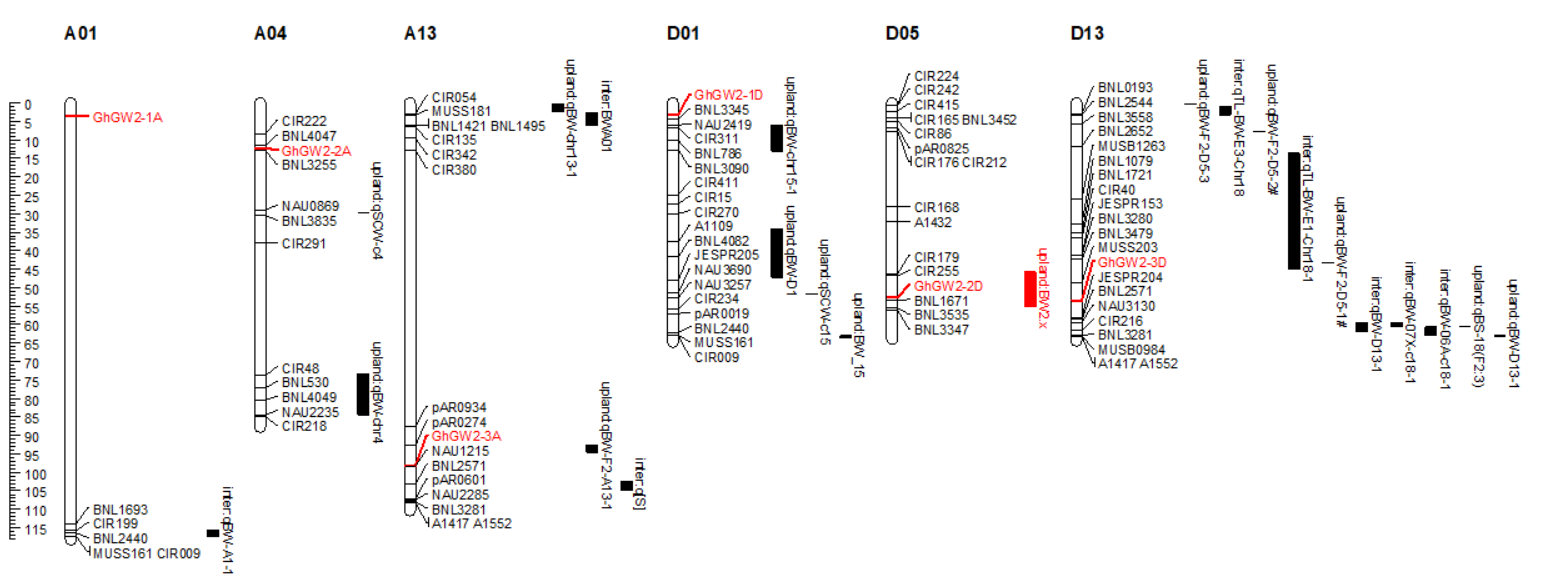

Supplement: Supplementary Figure 3 — A co-localization analysis of GhGW2-like genes with seed size quantitative trait loci (QTLs). GhGW2-like genes and co-localized QTL are shown in red. [file Image_3.JPEG]
